# Exploratory descriptive cross-sectional study on loneliness and participation in leisure activities among non-institutionalized older adults

**DOI:** 10.3389/fpubh.2026.1844240

**Published:** 2026-06-11

**Authors:** Blanca Gonzalez-Sánchez, María Macias-Quintero, Santiago Gómez-Paniagua, Juan Rodríguez-Mansilla, Elisa María Garrido-Ardila, María Jiménez-Palomares

**Affiliations:** 1 ADOLOR Research Group, Department of Medical-Surgical Therapy, Medicine Faculty and Health Sciences, University of Extremadura, Badajoz, Spain; 2 Occupational Therapist, Vitalia Alcosa Center, Sevilla, Spain; 3 Promoting a Healthy Society Research Group (PHeSO), Faculty of Sport Sciences, University of Extremadura, Cáceres, Spain

**Keywords:** ESTE II, leisure, loneliness, non-institutionalized, older (diseased) population

## Abstract

Loneliness is described as a painful experience caused by the absence of social relationships or feelings of belonging. Furthermore, it is currently considered one of the greatest challenges in the lives of older adults. The purpose of this study is to determine the level of perceived loneliness by the 89 participants, who are students at the University of Extremadura for Older Adults. To this end, we conducted an exploratory descriptive cross-sectional study, using the ESTE II perceived loneliness questionnaire as a measurement tool. This scale allows us to assess the social support, use of technology, and social participation of these individuals. The results obtained have allowed us to observe that there are no significant differences between the different variables. However, a moderate association has been established between levels of perceived loneliness and factors such as widowhood, having a large number of children, or a lack of social or cultural leisure activities. In conclusion, it has been determined that the participants showed a low level of perceived loneliness.

## 1 Introduction

In recent years, the issue of loneliness has become increasingly prevalent. For decades, loneliness has been associated with individuals lacking a defined family group or not belonging to a particular social environment. However, loneliness is currently recognized as a problem that affects all social strata, with older adults representing the most affected group (1). Recent international evidence confirms that loneliness affects a substantial proportion of the older population worldwide, with a pooled prevalence of 27.6%, reaching over 30% in North America and exceeding 50% among institutionalized older adults (18).

The high prevalence of loneliness among older adults has led to its characterization as one of the most devastating conditions affecting later life (1). According to data from the Social Observatory of the “la Caixa” Foundation, 68.4% of older adults report experiencing loneliness, underscoring the magnitude of the problem. In line with these findings, longitudinal studies have demonstrated that loneliness is associated with increased all-cause mortality, higher hospitalization rates, and greater use of healthcare services among older adults (2).

Loneliness is described in the literature as a painful experience resulting from the absence of social relationships or a lack of perceived belonging, and it is considered one of the greatest challenges faced by older adults (3). It may have a negative impact on their lives, as evidence suggests that loneliness can lead to adverse physical and mental health outcomes. Physically, it has been associated with immune system impairment, an increased risk of cardiovascular disease, and greater use of medications and healthcare services. In terms of mental health, loneliness may undermine self-esteem, increase the likelihood of depression, and reduce self-care behaviors (4).

Additional evidence has linked loneliness with higher prevalence of depressive symptoms, anxiety, and reduced psychological well-being in later life (5).

Social participation, emotional support, engagement in leisure activities, and the presence of a strong social network are recognized as protective factors against loneliness. These elements contribute to improved physical and mental health and may delay or prevent the onset of functional dependence (4). However, scientific evidence indicates that these associations are primarily associative rather than causal. Recent studies suggest that social participation, emotional support, and engagement in leisure activities are linked to better physical and mental health outcomes and a lower risk of functional decline among older adults, although the preventive effect on functional dependence should be interpreted with caution and supported by longitudinal evidence (6, 7). Recent literature highlights that community-based and socially oriented interventions combining social engagement and meaningful activity are particularly effective in reducing perceived loneliness among older adults (8).

Leisure is defined as “a non-obligatory activity that is intrinsically motivated and in which individuals engage during discretionary or free time, that is, time not committed to obligatory occupations such as work, self-care, or sleep” (9). From a preventive perspective, participation in group-based occupational workshops is recommended in order to promote active and meaningful leisure. Such activities stimulate social participation and expand social networks, thereby helping to prevent loneliness and social isolation (10). Importantly, recent research has identified key facilitators for older adults’ participation in leisure activities, including: perceived physical capability and enjoyment; safe and accessible environments; opportunities for meaningful social interaction; identity as an “exerciser”; and motivational aspects such as health aspirations and the presence of peer or family support (11–13).

In Spain, 36.8% of individuals aged 65 years and older live alone, and the prevalence of isolation and loneliness in this population ranges between 20 and 40%. Moreover, those residing in their own homes or in the home of a family member often have reduced access to structured support networks, which may increase the risk of experiencing loneliness and social isolation. This lack of regular social interaction can negatively affect both physical and mental well-being (14).

According to a United Nations report, by 2025 Spain will be the second most aged country in the world, with 35% of its population aged 65 years and older. This percentage is notably high when compared with other European countries, placing Spain among the most aged populations not only globally but also within the European context, where the proportion of older adults is generally lower. This comparison highlights the magnitude of population aging in Spain and underscores the demographic challenge posed by this trend. This demographic shift is associated with the growing number of individuals reaching older age, alongside an increase in the prevalence of older adults who are dependent or at risk of dependency (15).

In Spain, more than one million individuals live alone in their homes, and evidence indicates that the likelihood of living alone increases with age (16). Those who live alone tend to report higher levels of loneliness compared with individuals who live with others and receive social support (17). Recent evidence indicates that this difference is particularly pronounced among older adults; for example, a comprehensive scoping review reports that nearly 20% of community-dwelling older adults live alone and exhibit significantly higher loneliness scores than younger cohorts, highlighting older adults living alone as a population at elevated risk (18). Therefore, addressing loneliness among older people requires a multidisciplinary approach. Therefore, addressing loneliness among older adults requires a multidisciplinary approach.

Accordingly, the objective of this study is to analyze the level of loneliness experienced by non-institutionalized adults aged over 60 years and to examine the influence of participation in leisure activities on loneliness within this population.

## 2 Materials and methods

### 2.1 Study design

This study employed an exploratory observational, descriptive, cross-sectional design conducted on a sample of students enrolled in the University for Older Adults of Extremadura (UMEx). Therefore, no prior calculation of the sample size was carried out. Data were collected using the Social loneliness Scale ESTE II (19), applying a convenience sampling strategy. The study complied with all ethical requirements established in the Declaration of Helsinki (20) and with current data protection regulations (21) As this is a non-invasive study, based on anonymous data and with written informed consent obtained from all participants, formal approval from the Bioethics and Biosafety Committee of the University of Extremadura was not required. All participants provided written informed consent prior to their inclusion in the study.”

### 2.2 Study population

The sample for this study consisted of 89 individuals aged over 60 years. All participants were non-institutionalized, meaning they resided in their own homes and were not enrolled in any residential or long-term care services. The selected participants were students of the University for Older Adults of Extremadura (UMEx). UMEx is an active aging and lifelong learning initiative promoted by the University of Extremadura, designed to enhance social participation, cognitive stimulation, and community engagement among older adults. During the 2024–2025 academic year, UMEx enrolled approximately 3,524 students across eight campuses—including Cáceres, Badajoz, Mérida, and Plasencia—which highlights its role as a major social and educational platform for older citizens in Extremadura (22). The exact number of students at the Cáceres campus was not available. Participants were recruited using convenience sampling, which may introduce selection bias by including individuals already embedded in active social networks.

The following inclusion criteria were established:

- Being over 60 years of age.
- Being enrolled as a student at the University for Older Adults of Extremadura.
- Not being institutionalized in any type of care service.

The exclusion criteria were as follows:

- Being under 60 years of age.
- Being institutionalized in any type of service.
- Not being enrolled in any course at the University for Older Adults of Extremadura.

### 2.3 Procedure

Initial contact with the UMEx was made by phone to explain the study’s objective, the details of participation, and how the study would be conducted. Subsequently Data collection for this study was conducted on April 1 during regular class hours at UMEx. All participants were informed that their participation in the study was anonymous and voluntary and that no incentives would be offered. A structured, self-administered interview format was used, as participants were able to access the questionnaire independently through a QR code provided to them. The questionnaire was administered in a face-to-face, supervised setting during lessons, with trained staff on hand to answer questions and provide technical support to participants who needed assistance accessing the questionnaire via the QR code.

The assessment instrument employed was the Social loneliness Scale ESTE II, designed to measure the impact and level of perceived loneliness.

In addition, each participant completed a questionnaire specifically developed for this study, which gathered information such as date of birth, marital status, place of residence, number of children, and recreational activities performed during leisure time.

The categorization of leisure activities was grounded in a review of the literature on leisure and active aging in older adults, which highlights participation in physical, cognitive, manual, and social activities as key components of well-being and quality of life in later life (36–38). In the present study, leisure was operationally defined as the type of leisure-time activity that participants reported engaging in predominantly, without considering quantitative parameters such as frequency, duration, or intensity of participation.

Based on the activities reported by participants aged 65 years and older, an analytical categorization was established comprising four groups: sports-related, cultural, manipulative, and travel activities. This categorization followed predefined operational criteria adapted to the functional and occupational characteristics of aging and was consistent with participation domains described in the gerontological and occupational literature (Jopp and Hertzog, 2010; Wion et al., 2019).

Sports-related activities included those involving physical exercise and body movement, such as walking, gym-based exercise, swimming, or group sports. Cultural activities encompassed activities primarily focused on cognitive or artistic engagement, including reading, attending cultural events, listening to music, or participating in cultural workshops. Manipulative activities referred to manual or craft-based activities, such as handicrafts, sewing, or do-it-yourself activities. Travel activities included leisure pursuits involving trips or excursions outside the usual place of residence. Each participant was assigned to the category corresponding to the activity they reported performing most predominantly during their leisure time, understood as their main leisure activity, without objective quantification of frequency or intensity.

Finally, all participants signed an informed consent form outlining the purpose of the research and guaranteeing the confidentiality and anonymity of their data.

All data were collected by personnel external to the research team in order to minimize potential bias.

Variables: First, a sociodemographic questionnaire was specifically developed for this study in order to collect variables relevant to the analysis of perceived loneliness. This questionnaire gathered information on age, date of birth, marital status, place of residence, number of children, and participation in leisure activities during free time. The inclusion of these variables enabled a comprehensive assessment of potential factors associated with perceived loneliness among non-institutionalized older adults.

Then, data were collected using the Social loneliness Scale ESTE II (19), an instrument designed to measure the level and impact of loneliness. This scale has been validated both nationally and internationally as a reliable tool for identifying levels of perceived loneliness.

The scale consists of 15 items, each with three possible response options: “never,” “sometimes,” and “always.” These items assess three main factors: (1) individual perception of social support (items 1–8); (2) use of new technologies (items 9–11); and (3) social participation index (items 12–15).

The total score is obtained by summing the individual item scores, yielding a possible range from 0 to 30 points, with higher scores indicating greater levels of loneliness. The levels of loneliness are categorized as follows: low (0–10 points), moderate (11–20 points), and high (21–30 points).

### 2.4 Statistical analysis

Data analysis was conducted using IBM SPSS for Mac, version 23. In the initial stage, the Kolmogorov–Smirnov test was applied to assess whether continuous variables followed a normal distribution. As the assumption of normality was not met (*p* < 0.05), nonparametric statistical methods were employed. Also, procedures for addressing missing data were not required because every questionnaire included in the study was completed in full form.

Subsequently, the Kruskal–Wallis test was used to examine differences in the scores on the ESTE II scale and its dimensions between subgroups defined by marital status, number of children, and participation in leisure activities, with the level of statistical significance set at *p* < 0.05. Furthermore, for these comparisons, effect sizes were calculated using the eta-squared statistic, which can be classified as small (≥0.01), medium (≥0.06) or large (≥0.14) (23). In cases where statistically significant differences were identified using the Kruskal–Wallis test, *post hoc* multiple comparisons were performed using Dunn’s test in order to specifically identify which groups exhibited these differences.

In addition, the potential relationship between ESTE II scale scores, its dimensions and the participants’ age was analyzed using Spearman’s Rho test, exploring how this association might vary across the aforementioned subgroups. The level of statistical significance was established at *p* < 0.05. Correlation coefficients were interpreted according to the criteria proposed by Mondragón-Barrera (24), which establish the following ranges:

- (no correlation)
- 0.01–0.10 (low correlation)
- 0.11–0.50 (moderate correlation)
- 0.51–0.75 (considerable correlation)
- 0.76–0.90 (very high correlation)
- 0.91–1.00 (perfect correlation)

Similarly, in order to control for the possible effect of confounding variables on levels of perceived loneliness, a multiple linear regression analysis was performed using the total score obtained on the ESTE II scale as the dependent variable. Age was included as a continuous covariate, whilst marital status, number of children and type of leisure activities were included as categorical factors. Furthermore, potential multicollinearity among the independent variables was assessed using tolerance statistics and the variance inflation factor (VIF). The level of statistical significance was set at *p* < 0.05.

Finally, the internal consistency of the scale was examined using Cronbach’s alpha coefficient. To interpret the reliability results, the criteria proposed by Nunnally and Bernstein (25) were applied, considering the following values:

- 0.70 (poor)
- 0.71–0.90 (good)
- 0.91 (excellent)

### 2.5 Reference management

The review of the scientific literature related to the subject of this study was conducted using the PubMed and Cochrane Library Plus databases.

Bibliographic references were managed in accordance with the Vancouver style guidelines, using the reference management software Zotero.

## 3 Results

To obtain the study results, the potential relationships between ESTE II scale scores and participants’ age were examined. In addition, possible variations in perceived loneliness were analyzed in relation to the different variable subgroups. For this purpose, the level of statistical significance was set at *p* < 0.05.

Table 1 shows the sociodemographic characteristics of the 89 participants included in the study. In terms of marital status, the largest group consisted of married individuals (58.4%), followed by widowed participants (14.6%). Both single and divorced individuals accounted for the same proportion of the sample (13.5%).

**Table 1:** Sample’s characteristics (*N* = 89).

Variable	Categories	*N*	%
Marital status	Single	12	13.5
	Married	52	58.4
	Divorced	12	13.5
	Widowed	13	14.6
Number of children	0	15	16.9
	1	13	14.6
	2	41	46.1
	3	16	18.0
	4	4	4.5
Leisure activities	Sports	50	56.2
	Cultural	29	32.6
	Manipulative	3	3.4
	Travel	7	7.9
Variable		*M*	SD
Age		68.34	4.32

N, number; %, percentage; SD, standard deviation; M, mean.

Regarding the number of children, the most common category was participants with two children (46.1%), followed by those with three children (18.0%). In contrast, the least represented group was participants with four children (4.5%).

Regarding leisure activities, sports were the most common among participants, with 56.2% of the sample engaging in them. Cultural activities accounted for 32.6% of the responses, while crafts and travel showed lower participation rates (3.4 and 7.9%, respectively).

The mean age of the participants was 68.34 years (SD = 4.32), with a narrow distribution of data around the mean.

**Figure 1:**
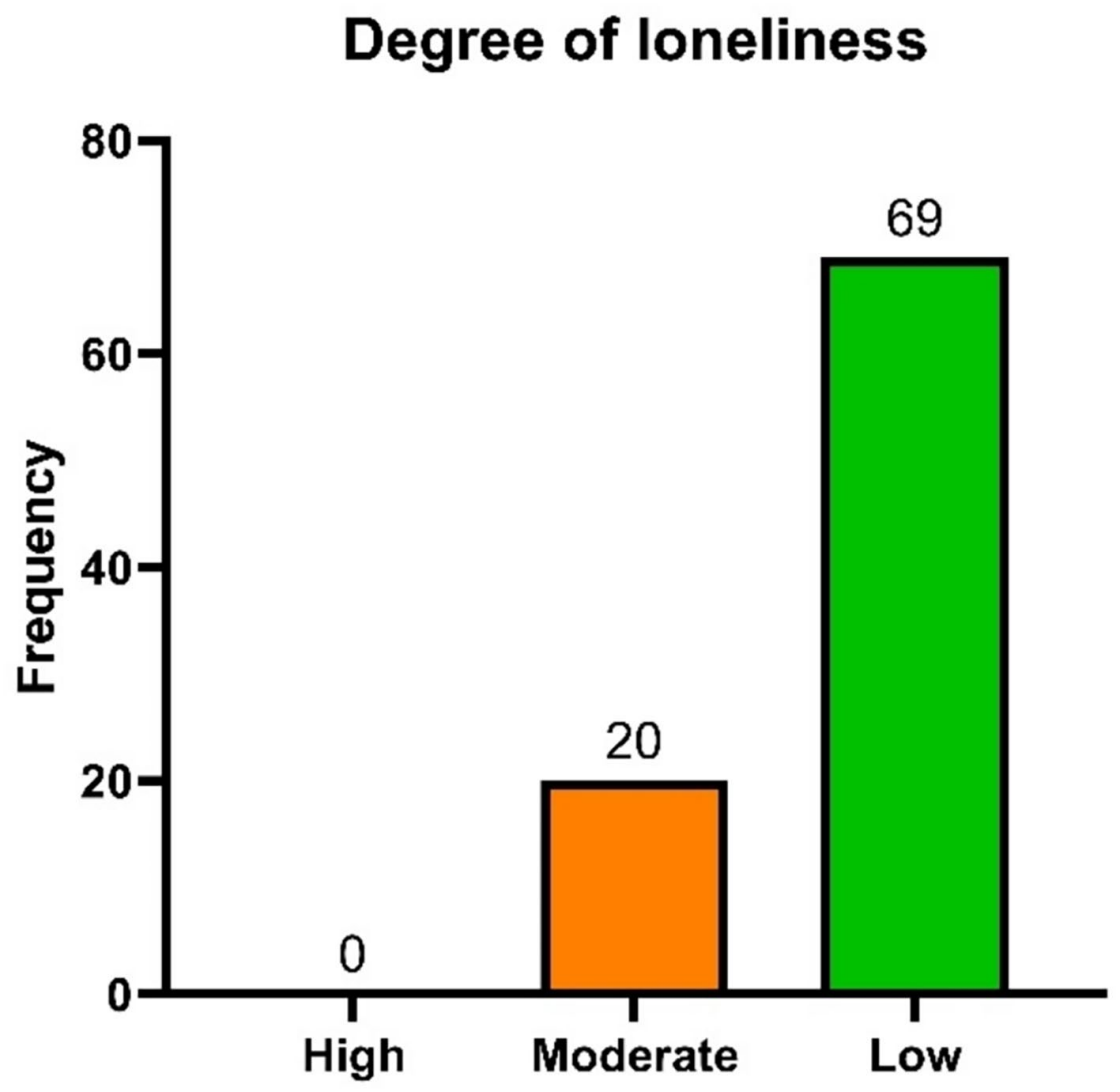
Frequency of the degree of loneliness.

**Figure 2:**
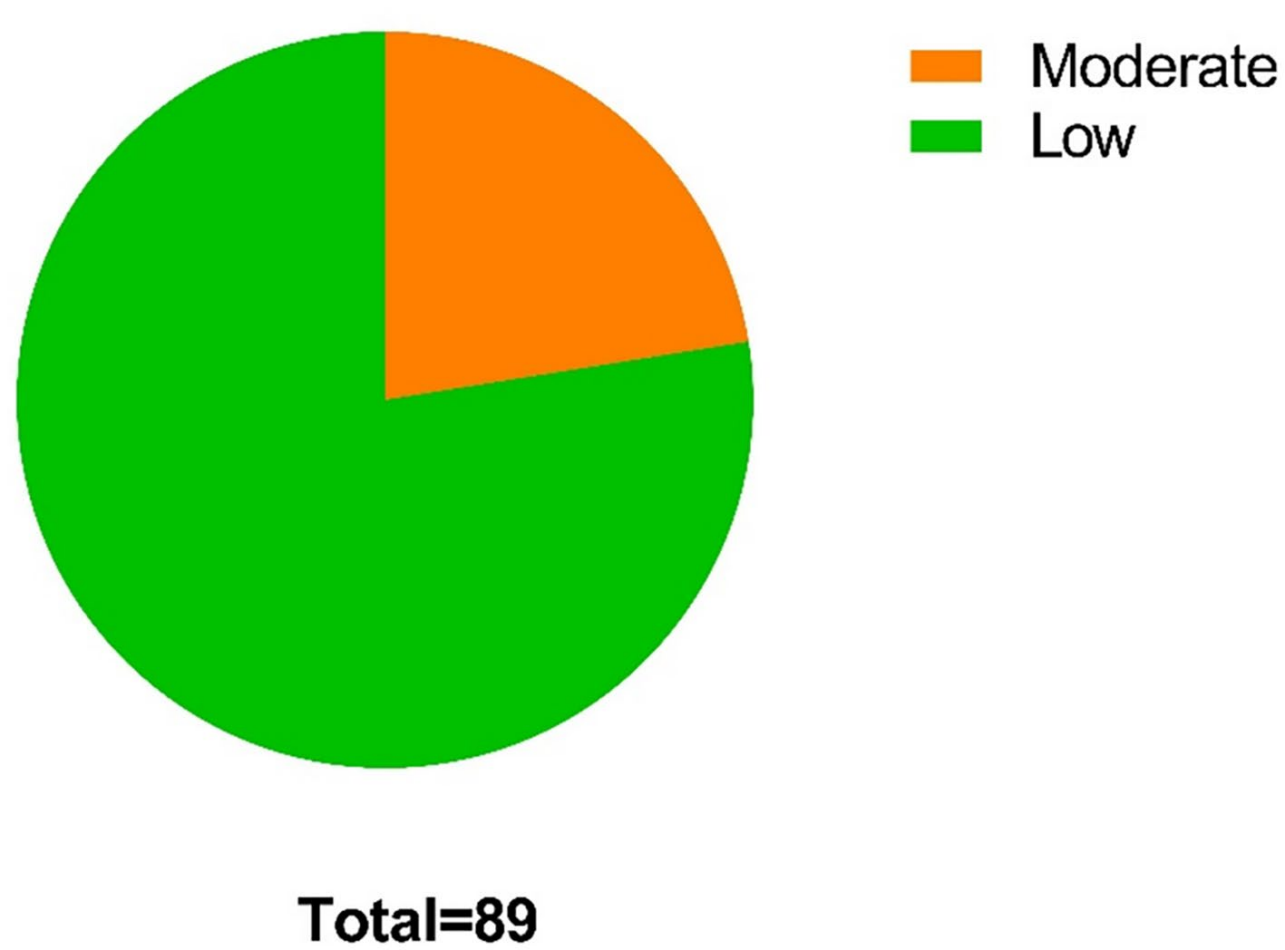
Distribution of loneliness levels.

The distribution of perceived loneliness levels is presented in Figures 1, 2. In absolute terms, 69 of the 89 participants were classified as having low levels of perceived loneliness, while 20 participants had moderate levels. No cases of high perceived loneliness were recorded.

In relative terms, 77.53% of the sample showed low levels of perceived loneliness, compared to 22.47% who exhibited moderate levels. These results indicate that, although most participants reported low levels of perceived loneliness, a significant proportion of the sample experienced moderate levels.

The results of the analysis of marital status and its relationship to perceived loneliness are shown in Table 2. No statistically significant differences were observed in the total score on the ESTE II scale among the different groups analyzed (*p* = 0.58). However, the group of widowed participants had the highest mean score compared to the other categories.

**Table 2:** Descriptive results of the ESTE II scale according to marital status.

Loneliness	Marital status					
	Single *M* (SD)	Married *M* (SD)	Divorced *M* (SD)	Widowed *M* (SD)	*p*	$\eta^{2}$
ESTE II	7.8 (2.6)	7.8 (3.2)	8.0 (3.6)	9.3 (3.2)	0.58	<0.01
Social support	3.0 (1.4) ^ab^	2.6 (2.0) ^a^	4.3 (2.0) ^b^	4.4 (2.1) ^b^	<0.01*	0.11
Technology use	1.6 (1.1)	1.6 (1.2)	1.1 (1.2)	1.3 (1.2)	0.56	<0.01
Social participation	3.2 (1.4)	3.6 (1.5)	2.7 (1.4)	3.6 (1.7)	0.23	0.02

M, mean; SD, standard deviation. Differences are statistically significant at *p < 0.05. Different superscript letters indicate statistically significant differences between groups.

Regarding the specific dimensions of the scale, statistically significant differences were identified only in the social support dimension (*p* < 0.01; η^2^ = 0.11). *Post hoc* analysis showed higher scores among divorced and widowed participants compared to married individuals. No significant differences were found in the dimensions of technology use or social participation.

Table 3 presents the results obtained based on the number of children. No statistically significant differences were observed in the total score on the ESTE II scale among the different groups analyzed (*p* = 0.66). Similarly, no significant differences were identified in the dimensions of social support, technology use, and social participation.

**Table 3:** Descriptive results of the ESTE II scale according to number of children.

Loneliness	Number of children						
	0 M (SD)	1 M (SD)	2 M (SD)	3 M (SD)	4 M (SD)	*p*	$\eta^{2}$
ESTE II	7.7 (2.4)	7.6 (3.4)	7.9 (3.7)	8.9 (2.3)	8.5 (3.1)	0.66	<0.01
Social support	3.3 (1.4)	2.8 (1.3)	3.2 (2.5)	3.2 (1.8)	2.8 (2.9)	0.94	<0.01
Technology use	1.4 (1.1)	1.8 (1.4)	1.3 (1.2)	1.8 (0.9)	1.3 (0.9)	0.48	<0.01
Social participation	3.1 (1.4)	3.0 (1.6)	3.4 (1.7)	3.9 (1.2)	4.5 (0.6)	0.17	0.03

M, mean; SD, standard deviation. Differences are statistically significant at *p < 0.05.

Nevertheless, participants with three children had the highest mean score on the ESTE II scale compared to the other groups. Regarding effect sizes, small values were observed across all dimensions analyzed (η^2^ < 0.06).

The results of the analysis of leisure activities are shown in Table 4. Statistically significant differences were observed in the total score on the ESTE II scale according to the type of leisure activity engaged in by the participants (*p* < 0.01; η^2^ = 0.17). *Post hoc* analysis revealed significantly higher scores among participants who engaged in cultural activities compared to those who participated in sports or traveled.

**Table 4:** Descriptive results of the ESTE II scale according to leisure activities.

Loneliness	Leisure activities					
	Sport *M* (SD)	Cultural *M* (SD)	Manipulative *M* (SD)	Travel *M* (SD)	*p*	$\eta^{2}$
ESTE II	7.1 (2.9) ^a^	9.9 (2.9) ^b^	9.7 (2.5) ^ab^	6.3 (2.5) ^a^	<0.01*	0.17
Social support	2.7 (1.8) ^a^	4.2 (2.3) ^b^	3.0 (2.0) ^ab^	2.6 (1.9) ^ab^	0.03*	0.07
Technology use	1.4 (1.2)	1.8 (1.1)	1.7 (1.2)	0.6 (0.8)	0.07	0.05
Social participation	3.1 (1.5) ^a^	3.9 (1.5) ^b^	5.0 (1.0) ^b^	3.1 (0.7) ^ab^	0.02*	0.08

M, Mean; SD, standard deviation. Differences are statistically significant at * *p* < 0.05.

Different superscript letters indicate statistically significant differences between groups.

Likewise, statistically significant differences were identified in the dimensions of social support (*p* = 0.03; η^2^ = 0.07) and social participation (*p* = 0.02; η^2^ = 0.08). In both cases, participants who engaged in cultural activities had higher scores than those who participated in sports. No statistically significant differences were observed in the dimension related to technology use (*p* = 0.07; η^2^ = 0.05).

The correlations between age and scores on the ESTE II scale and its dimensions are presented in Table 5. In general, no statistically significant correlations were observed between age and levels of perceived loneliness in the total sample analyzed.

**Table 5:** Correlations between ESTE II scale scores and age according to study subgroups.

Variable	Subgroup	ESTE II *ρ (p)*	Social support *ρ (p)*	Technology use *ρ (p)*	Social participation *ρ (p)*
Age	Total	0.01 (0.90)	0.02 (0.84)	0.05 (0.64)	0.03 (0.81)
Marital status	Single	0.01 (0.98)	−0.09 (0.78)	0.19 (0.55)	0.04 (0.98)
	Married	−0.01 (0.48)	−0.06 (0.63)	−0.01 (0.96)	−0.07 (0.64)
	Divorced	−0.03 (0,91)	−0.04 (0.91)	−0.09 (0.76)	0.29 (0.37)
	Widowed	0.27 (0.38)	0.34 (0.25)	0.37 (0.12)	−0.10 (0.74)
Number of children	0	−0.09 (0.74)	0.01 (0.96)	−0.13 (0.65)	−0.06 (0.83)
	1	−0.11 (0.72)	0.04 (0.89)	−0.21 (0.49)	−0.30 (0.33)
	2	0.04 (0.81)	0.10 (0.55)	0.10 (0.56)	0.01 (0.99)
	3	0.01 (0.99)	0.01 (0.99)	−0.04 (0.88)	−0.06 (0.83)
	4	0.95 (0.05)	0.99 (<0.01*)	−0.50 (0.50)	0.71 (0.29)
Leisure activities	Sport	0.09 (0,54)	0.06 (0.69)	0.10 (0.51)	0.06 (0.70)
	Cultural	−0.04 (0.85)	−0.02 (0.93)	0.06 (0.75)	0.05 (0.79)
	Manipulative	−0.50 (0.67)	0.50 (0.99)	−0.86 (0.33)	−0.99 (0.33)
	Travel	−0.31 (0.54)	−0.15 (0.75)	0.12 (0.79)	−0.66 (0.11)

Differences are statistically significant at *p < 0.05.

Similarly, no statistically significant associations were identified between age and ESTE II scale scores in the various subgroups defined by marital status, number of children, or type of leisure activity, except in the group of participants with four children, where a high positive correlation was observed between age and the social support dimension (*ρ* = 0.99; *p* < 0.01).

The results of the multiple linear regression analysis are presented in Table 6. The model explained 21% of the variance observed in the total scores on the ESTE II scale (R^2^ = 0.21), although the overall model did not reach statistical significance (*F* = 1.864; *p* = 0.06). In this sense, only the type of leisure activity showed a statistically significant association with levels of perceived loneliness. Specifically, participation in cultural activities was associated with higher scores on the ESTE II scale compared with sporting activities (*B* = 2.709; *p* < 0.01). No statistically significant associations were observed for the remaining variables included in the model. In addition, multicollinearity analyses revealed no significant issues among the independent variables included in the model, with all VIF values being less than 2.

**Table 6:** Multiple linear regression model for perceived loneliness.

Model 1 (R^2^) = 0.21				
Variable	*B*	SE	*t*	*p*
Age	0.04	0.09	0.39	0.70
Married	0.29	2.04	0.14	0.89
Divorced	0.38	2.23	0.17	0.87
Widowed	1.08	2.15	0.50	0.62
One child	−0.12	1.99	−0.06	0.95
Two children	−0.54	1.88	−0.29	0.78
Three children	0.02	1.95	0.01	0.99
Four children	0.13	2.35	0.05	0.96
Cultural leisure	2.71	0.75	3.61	<0.01*
Manipulative leisure	2.32	1.86	1.25	0.22
Travel leisure	−0.89	1.32	−0.68	0.50
Constant	4.64	6.19	0.75	0.46

Reference categories: single marital status, no children, and sports-related leisure activities. Differences are statistically significant at *p < 0.05.

Finally, the scale yielded a Cronbach’s alpha value of 0.71, which, according to the literature, can be classified as good.

## 4 Discussion

The results of this study indicate that a high percentage of participants (77.53%) experience low levels of perceived loneliness. When comparing these findings with the general Spanish context, a significant discrepancy emerges. For instance, the Fundación “la Caixa” (4) reported that 68.4% of older adults in Spain experience feelings of loneliness, a figure notably higher than the one found in our sample at the University for Older Adults of Extremadura (UMEx).

However, it is imperative to address the conceptual and statistical limitations regarding the sample. As we are dealing with university students, there is an inherent selection bias; these individuals tend to have higher social motivation and cognitive reserve. This “healthy participant effect” suggests that while the UMEx environment acts as a protective micro-community, aligning with the theories of Rubio Herrera et al. (35), the results may not be directly generalizable to more vulnerable populations, such as institutionalized older adults or those with lower educational attainment. This distinction is crucial to avoid controversial over-applications of the data.

The significant difference found in loneliness levels according to the type of leisure activity (*p* < 0.01) allows for a deeper delve into how free time is utilized. Our data reveals that participants engaged in sports and travel reported lower loneliness than those in cultural activities.

This can be interpreted through the lens of social reciprocity. While cultural activities (reading, attending seminars) are intellectually enriching, they can often be “contemplative” or solitary. In contrast, sports and travel are high-interaction modalities that require constant interpersonal coordination. This finding converges with Pinquart and Sörensen (32), who established that the quality and nature of social engagement are stronger predictors of emotional well-being than the mere quantity of activities. Furthermore, following Nyqvist et al. (26), we argue that the “active ingredients” of leisure are shared presence and synchronous communication, which are more prevalent in physical and group-based occupations.

Regarding marital status, although global statistical significance was not reached (*p* = 0.58), the descriptive higher scores among widowed participants align with the “Social Convoy” model. As highlighted by Rodríguez-Borlado (27), the loss of a spouse represents the collapse of the primary attachment figure, a gap that institutional leisure can mitigate but not always fully replace.

A particularly interesting finding is the paradox of family size: participants with three children reported higher loneliness scores. This challenges traditional views but supports recent studies (33) suggesting that in modern society, loneliness is often a product of an “expectation gap.” Older adults with larger families may expect a level of interaction that busy modern lifestyles cannot fulfill, leading to a deeper sense of perceived abandonment compared to those with smaller, more realistic social networks.

To ensure the practical application of these results and reduce the risk of controversy, public policies must be tailored to the functional diversity of the population, moving toward a model of “Social Prescribing” (33).

- *For active older adults*: Incentives should focus on maintaining high-interaction activities to prevent the “expectation gap” and social atrophy. We propose “Social Mentorship” programs where active students lead travel clubs or sports teams. Following the timing of social vulnerability, these should be scheduled during evening hours or weekends to provide structure to time traditionally associated with higher loneliness in non-institutionalized populations (26).
- *For individuals with morbidity or functional disability*: The modality must shift toward inclusive and adapted leisure to ensure equity in access to social health. We recommend community-based “satellite” workshops in accessible neighborhood settings, scheduled during morning hours to align with higher energy levels and better safety conditions.
- *Settings and incentives*: Public health strategies should integrate Occupational Therapists to adapt settings—such as inclusive cultural tours or adapted group exercises—ensuring that physical limitations do not lead to social withdrawal (34). Furthermore, incentives such as “Social Vouchers” for travel and sports should specifically target widowed individuals or those living alone, facilitating their entry into the protective networks identified in the ESTE II framework (Rubio Herrera et al., 2011)

### 4.1 Limitation

The use of a convenience sample recruited from the University of the Third Age (UMEx) may have introduced selection bias, potentially leading to an underestimation of perceived loneliness and limiting the generalizability of the results. As participants were already involved in an active educational and social environment, their levels of perceived loneliness may differ from those of the general older population. In addition, the lack of prior sample size calculation and effect size estimation constitutes a further limitation of this study. Given that the analyses were primarily descriptive and exploratory in nature, the results should be interpreted with caution. Future studies employing analytical designs, larger and more diverse samples, and appropriate sample size calculations are needed to allow estimation of effect sizes and to draw more robust inferential conclusions. Furthermore, the use of a self-administered questionnaire accessed via a QR code may have favored the participation of individuals with higher levels of digital literacy, thereby introducing an additional source of potential selection bias.

Finally, it is important to acknowledge that the cross-sectional design of this study limits our ability to establish causality. While significant associations were found between specific leisure activities and lower levels of loneliness, these results should be interpreted as correlations. Future longitudinal research is required to determine the causal pathways between university-based social participation and the reduction of perceived loneliness over time.

### 4.2 Future lines of research

To address the limitations of this exploratory study, future research should:

- *Expand sample diversity*: Include older adults from rural vs. urban areas of Extremadura to compare how the environment influences the effectiveness of UMEx.
- *Longitudinal tracking*: Follow UMEx students over several years to determine if continued university participation can reverse pre-existing states of chronic loneliness.
- *Qualitative depth*: Implement semi-structured interviews to understand the “lived experience” of loneliness behind the ESTE II scores, particularly regarding why cultural leisure is perceived as more solitary.
- *Interventional studies*: Design and test specific “Leisure-Based Occupational Programs” that prioritize high-interaction activities (like team sports) to see if they reduce loneliness scores more effectively than standard educational programs
- Analyze the impact of loneliness in diverse socioeconomic strata to mitigate the current sample bias.
- Study the effectiveness of digital-social leisure for those with physical mobility restrictions.

### 4.3 Practical implications

The evidence gathered leads to several clinical and social recommendations:

- *For occupational therapists*: When prescribing leisure, it is not enough to “keep the person busy.” Interventions must prioritize high-interaction occupations (travel, group exercise) for those showing high scores on the ESTE II scale.
- *For UMEx administration*: Introduce “social mentoring” programs where veteran students welcome new ones, specifically targeting widowed individuals to facilitate their integration.
- *For public health*: Loneliness should be treated as a clinical vital sign. The ESTE II scale should be integrated into primary care screenings for non-institutionalized older adults to allow for “social prescribing” (referring patients to university or community groups).
- *For families*: Awareness campaigns are needed to explain that “being there” is not enough; the quality of the interaction and the reduction of the expectation gap are key to the emotional well-being of their elders.

## 5 Conclusion

No statistically significant results were found that directly influence levels of loneliness among non-institutionalized adults aged over 60 years. However, certain factors, such as widowhood and having three children, appear to be associated with a higher prevalence of perceived loneliness.

Differences in perceived loneliness were observed according to the type of leisure activities performed by participants, with lower loneliness scores reported by those engaged in sports activities or travel. These findings should be interpreted within the context of the studied population and do not imply causal relationships.

Individuals who engaged in sports activities or travel reported lower levels of loneliness compared with those who participated primarily in cultural activities.

## Data Availability

The raw data supporting the conclusions of this article will be made available by the authors, without undue reservation.
