# Supplementary material for: Exploratory descriptive cross-sectional study on loneliness and participation in leisure activities among non-institutionalized older adults
Source: Front Public Health. 2026 Jun 11;14:1844240. doi: 10.3389/fpubh.2026.1844240 (PMC13293820; doi:10.3389/fpubh.2026.1844240)
Supplement: Supplementary file 1 [file Data_Sheet_1.PDF]

SOCIODEMOGRAPHIC QUESTIONNAIRE

PERSONAL IDENTIFICATION

1. **FULL NAME** (First and Last Name)
2. **DATE OF BIRTH**
3. **CITY / LOCATION**
4. **MARITAL STATUS**
5. **DO YOU HAVE CHILDREN? IF SO, HOW MANY?**
6. **WHAT ACTIVITIES DO YOU PERFORM IN YOUR LEISURE TIME?**
